# LMS parameters, percentile, and Z-score growth curves for axial length in Chinese schoolchildren in Wuhan

**DOI:** 10.1038/s41598-022-08907-5

**Published:** 2022-03-22

**Authors:** Pablo Sanz Diez, Li-Hua Yang, Mei-Xia Lu, Wieland Kiess, Siegfried Wahl

**Affiliations:** 1 Institute for Ophthalmic Research, Eberhard Karls University Tuebingen, Elfriede-Aulhorn-Strasse 7, 72076 Tuebingen, Germany; 2 Carl Zeiss Vision International GmbH, Technology and Innovation, Turnstrasse 27, 73430 Aalen, Germany; 3 Wuhan Center for Adolescent Poor Vision Prevention and Control, Wuhan, China; 4 Wuhan Commission of Experts for the Prevention and Control of Adolescent Poor Vision, Wuhan, China; 5 Department of Epidemiology and Statistics, School of Public Health, Tongji Medical College, Huazhong University of Science and Technology, Wuhan, China; 6 Leipzig Research Center for Civilization Diseases (LIFE), Leipzig University, Leipzig, Germany; 7 Department of Women and Child Health, University Hospital for Children and Adolescents and Center for Pediatric Research, Leipzig University, Leipzig, Germany; 8 Center for Pediatric Research, Leipzig University, Leipzig, Germany

**Keywords:** Paediatrics, Public health

## Abstract

Understanding the ocular structural changes are fundamental to defining strategies for myopia prevention and management. This study aimed to establish age-gender specific normative LMS parameters for axial length to generate percentile and Z-score growth curves in a population of Chinese schoolchildren. A total of 14,760 individuals aged 6 to 15 years from Wuhan, central China, contributed to this study. The LMS method was used for the calculation of LMS parameters and the generation of percentile and Z-score growth curves for axial length. Growth curves derived from the LMS parameters were compared with those originally calculated. Axial elongation was age- and percentile-dependent. The highest elongation rate occurred at the 98th percentile in the range 6 to 9 years, being up to 1.46 mm in boys and 1.42 mm in girls. The largest differences between original and newly generated growth curves were detected at the 98th percentile at age 15; 0.78 mm (females) and 0.63 mm (males). Multinomial logistic regression and receiver operating characteristic analyses revealed Z-scores as a good predictor for estimating high myopia development. The axial length growth curves presented in this study provide a technically solid instrument that depicts the best description of physiological eye growth for Chinese schoolchildren aged 6 to 15 years.

## Introduction

Normative growth curves are an essential tool in pediatric primary health care. They help to assess the physiological characteristics and development of children according to age, gender, and ethnicity^1,2^. Since its appearance more than two hundred years ago^3^, numerous charts have been generated for different anthropometric variables^4–6^. Over decades, growth chart development has been focused on the assessment of nutritional status and growth of children through anthropometric variables such as height, weight, body circumferences and skinfold thickness^1,2,7^. However, scientific publications on growth curves derived from ocular variables have been scarce and recent. Concretely, axial length and refractive error have been the most common variables used to generate ocular growth curves^8–15^.

The construction of growth references and charts involves several aspects such as gathering specific anthropometric data for a given age range and population, statistical data analysis and fitting methods and chart graphical representation^3^. Statistical tools such as percentiles and Z-scores have been widely used to generate and describe growth curves. Percentile indicates the percentage of observations that fall below a certain value, while Z-score represents the distance and direction of an observation away from the population median^16^. In comparison to Z-score, percentiles are more widespread since they are easier to understand and use in clinical practice^16^. Several mathematical methods have been described for attained percentiles and Z-scores curves^17^, which mainly differ in the treatment of age as grouping variable and in the assessment of distributional assumptions^17^. One of the most well-known is the LMS method (Lambda Mu and Sigma method), which enables efficient calculation of percentiles and Z-scores and smoothing of growth curves^18–20^. The LMS method was first developed by Cole^19,20^ and later extended by Cole and Green^18^. The LMS method is based on the use of Box–Cox transformations^21^ to normality through the calculation of a skewness parameter^22^. Given its efficiency and flexibility it has been applied as a standard method in many benchmark studies^1,23–25^. Particularly, LMS technique has not been previously used for the generation of growth curves for ocular variables, such as axial length. Therefore, in this paper, we aim at demonstrating the application of the LMS method for population-based ocular axial length data. We report the basis for the calculation of LMS parameters and the subsequent estimation of axial length growth curves in a Chinese school-age population. In addition, the new percentile growth curves derived from the LMS parameters are compared with the original percentile curves previously calculated and published from the same study population.

## Results

### LMS parameters

Table 1 shows the different combinations of edf values chosen for the modelling and optimization process of the new calculated percentile curves. The edf values were increased and reduced one at a time until the smallest SBC value was found. First, we set the initial edf values for L to 0 and for M and S to 1. Considering edf (M) > edf (S) > edf (L), edf values for M were increased first, followed by edf values for S. While higher edf values for M and S generated smaller SBC values, greater edf values for L produced higher SBC values (Table 1). For both genders, the best model optimization was achieved with edf values of L = 0, M = 4, and S = 2, as they provided the highest reduction in SBC and a reasonable smoothing. As seen in Table 1, a subsequent increase in edf values did not lead to a reduction in SBC values. Tables 2 and 3 show the LMS parameters for axial length. In both females and males, value of 1 was used to represent the L curve at all ages. M curve increased with age in both genders. On average, males presented higher but not significantly different than females (average difference = 0.54 ± 0.04 mm; p = 0.10, t-value =  − 1.75, df = 18; two-sample t-test). S curve increased with age in both genders, reaching the highest values at age 15. Both groups showed similar S curves (p = 0.81, t-value = 0.24, df = 18; two-sample t-test).

**Table 1:** Sequences of equivalent degrees of freedom (edf) chosen for L, M and S curve optimization process in females and males.

Equivalent degrees of freedom			Female		Male	
edf (L)	edf (M)	edf (S)	SBC^a^	Change in SBC^b^	SBC^a^	Change in SBC^b^
0	1	1	17,880.4	–	19,568.3	–
1	1	1	17,884.8	+ 4.4	19,576.2	+ 7.9
1	2	1	16,060.6	− 1824.2	17,424.9	− 2151.3
1	2	2	16,049.4	− 11.2	17,415.9	− 9.0
2	2	2	16,052.4	+ 3.0	17,416.0	+ 0.1
1	3	2	15,836.0	− 216.4	17,173.9	− 242.1
2	3	2	15,841.3	+ 5.3	17,178.0	+ 4.1
1	3	3	15,836.7	− 4.6	17,178.2	+ 0.2
0	4	2	15,824.7	− 12.0	17,158.9	− 19.3
1	4	2	15,832.9	+ 8.2	17,165.7	+ 6.8
1	4	3	15,833.4	+ 0.5	17,170.3	+ 4.6
2	4	3	15,836.8	+ 3.4	17,173.0	+ 2.7

^a^Schwarz Bayesian Criterion (SBC) was used as a deviance measure for checking the goodness of fit.

^b^Change in SBC indicates the corresponding change in deviance values with the previous SBC value.

**Table 2:** LMS parameters and axial length percentiles in millimeters, as a function of age and gender.

Age	L	M	S	Percentiles (axial length in mm)								
				2nd	5th	10th	25th	50th	75th	90th	95th	98th
**Female**												
6	1.0	22.52	0.0362	20.84	21.18	21.47	21.97	22.52	23.07	23.56	23.86	24.19
7	1.0	22.94	0.0372	21.19	21.54	21.85	22.37	22.94	23.52	24.04	24.35	24.70
8	1.0	23.37	0.0381	21.54	21.90	22.22	22.77	23.37	23.97	24.51	24.83	25.20
9	1.0	23.71	0.0389	21.82	22.19	22.53	23.09	23.71	24.34	24.90	25.23	25.61
10	1.0	23.94	0.0395	22.00	22.38	22.73	23.30	23.94	24.58	25.15	25.49	25.88
11	1.0	24.09	0.0399	22.12	22.51	22.86	23.44	24.09	24.73	25.32	25.67	26.06
12	1.0	24.21	0.0402	22.21	22.61	22.96	23.55	24.21	24.87	25.46	25.81	26.21
13	1.0	24.32	0.0405	22.30	22.70	23.06	23.66	24.32	24.98	25.58	25.94	26.34
14	1.0	24.41	0.0407	22.37	22.77	23.13	23.74	24.41	25.08	25.68	26.04	26.45
15	1.0	24.49	0.0409	22.43	22.84	23.21	23.82	24.49	25.17	25.78	26.14	26.55
**Male**												
6	1.0	22.98	0.0363	21.27	21.61	21.91	22.42	22.98	23.54	24.05	24.35	24.70
7	1.0	23.42	0.0372	21.63	21.99	22.31	22.84	23.42	24.01	24.54	24.86	25.21
8	1.0	23.87	0.0381	22.00	22.37	22.70	23.25	23.87	24.48	25.03	25.36	25.73
9	1.0	24.23	0.0388	22.30	22.69	23.03	23.60	24.23	24.86	25.43	25.78	26.16
10	1.0	24.48	0.0393	22.50	22.89	23.24	23.83	24.48	25.12	25.71	26.06	26.45
11	1.0	24.64	0.0397	22.64	23.04	23.39	23.98	24.64	25.30	25.89	26.25	26.65
12	1.0	24.78	0.0400	22.75	23.15	23.51	24.11	24.78	25.45	26.05	26.41	26.81
13	1.0	24.90	0.0402	22.84	23.25	23.61	24.22	24.90	25.57	26.18	26.54	26.95
14	1.0	24.98	0.0404	22.91	23.32	23.69	24.30	24.98	25.66	26.28	26.64	27.05
15	1.0	25.07	0.0406	22.98	23.39	23.76	24.38	25.07	25.75	26.37	26.74	27.16

**Table 3:** LMS parameters and axial length Z-scores in millimeters, as a function of age and gender.

Age	L	M	S	Z-scores (axial length in mm)												
				− 3.0 SD	− 2.5 SD	− 2.0 SD	− 1.5 SD	− 1.0 SD	− 0.5 SD	0.0 SD	+ 0.5 SD	+ 1.0 SD	+ 1.5 SD	+ 2.0 SD	+ 2.5 SD	+ 3.0 SD
**Female**																
6	1.0	22.52	0.0362	20.07	20.48	20.89	21.30	21.70	22.11	22.52	22.93	23.33	23.74	24.15	24.56	24.97
7	1.0	22.94	0.0372	20.38	20.81	21.24	21.66	22.09	22.52	22.94	23.37	23.80	24.22	24.65	25.08	25.50
8	1.0	23.37	0.0381	20.69	21.14	21.58	22.03	22.48	22.92	23.37	23.81	24.26	24.70	25.15	25.60	26.04
9	1.0	23.71	0.0389	20.94	21.41	21.87	22.33	22.79	23.25	23.71	24.17	24.64	25.10	25.56	26.02	26.48
10	1.0	23.94	0.0395	21.10	21.58	22.05	22.52	22.99	23.47	23.94	24.41	24.88	25.36	25.83	26.30	26.77
11	1.0	24.09	0.0399	21.21	21.69	22.17	22.65	23.13	23.61	24.09	24.57	25.05	25.53	26.01	26.49	26.97
12	1.0	24.21	0.0402	21.29	21.78	22.27	22.75	23.24	23.72	24.21	24.70	25.18	25.67	26.16	26.64	27.13
13	1.0	24.32	0.0405	21.37	21.86	22.35	22.84	23.34	23.83	24.32	24.81	25.31	25.80	26.29	26.78	27.27
14	1.0	24.41	0.0407	21.43	21.92	22.42	22.92	23.41	23.91	24.41	24.90	25.40	25.90	26.39	26.89	27.38
15	1.0	24.49	0.0409	21.49	21.99	22.49	22.99	23.49	23.99	24.49	24.99	25.49	25.99	26.49	27.00	27.50
**Male**																
6	1.0	22.98	0.0363	20.48	20.89	21.31	21.73	22.15	22.56	22.98	23.40	23.82	24.23	24.65	25.07	25.49
7	1.0	23.42	0.0372	20.81	21.24	21.68	22.12	22.55	22.99	23.42	23.86	24.30	24.73	25.17	25.60	26.04
8	1.0	23.87	0.0381	21.14	21.59	22.05	22.50	22.96	23.41	23.87	24.32	24.77	25.23	25.68	26.14	26.59
9	1.0	24.23	0.0388	21.41	21.88	22.35	22.82	23.29	23.76	24.23	24.70	25.17	25.64	26.11	26.58	27.05
10	1.0	24.48	0.0393	21.59	22.07	22.55	23.03	23.51	24.00	24.48	24.96	25.44	25.92	26.40	26.88	27.36
11	1.0	24.64	0.0397	21.71	22.20	22.69	23.18	23.67	24.15	24.64	25.13	25.62	26.11	26.60	27.09	27.57
12	1.0	24.78	0.0400	21.81	22.31	22.80	23.30	23.79	24.29	24.78	25.28	25.77	26.27	26.76	27.26	27.75
13	1.0	24.90	0.0402	21.89	22.39	22.89	23.39	23.89	24.40	24.90	25.40	25.90	26.40	26.90	27.40	27.90
14	1.0	24.98	0.0404	21.95	22.46	22.96	23.47	23.97	24.48	24.98	25.49	25.99	26.50	27.00	27.51	28.01
15	1.0	25.07	0.0406	22.02	22.52	23.03	23.54	24.05	24.56	25.07	25.58	26.09	26.59	27.10	27.61	28.12

### Percentile growth curves derived from LMS parameters

**Figure 1:**
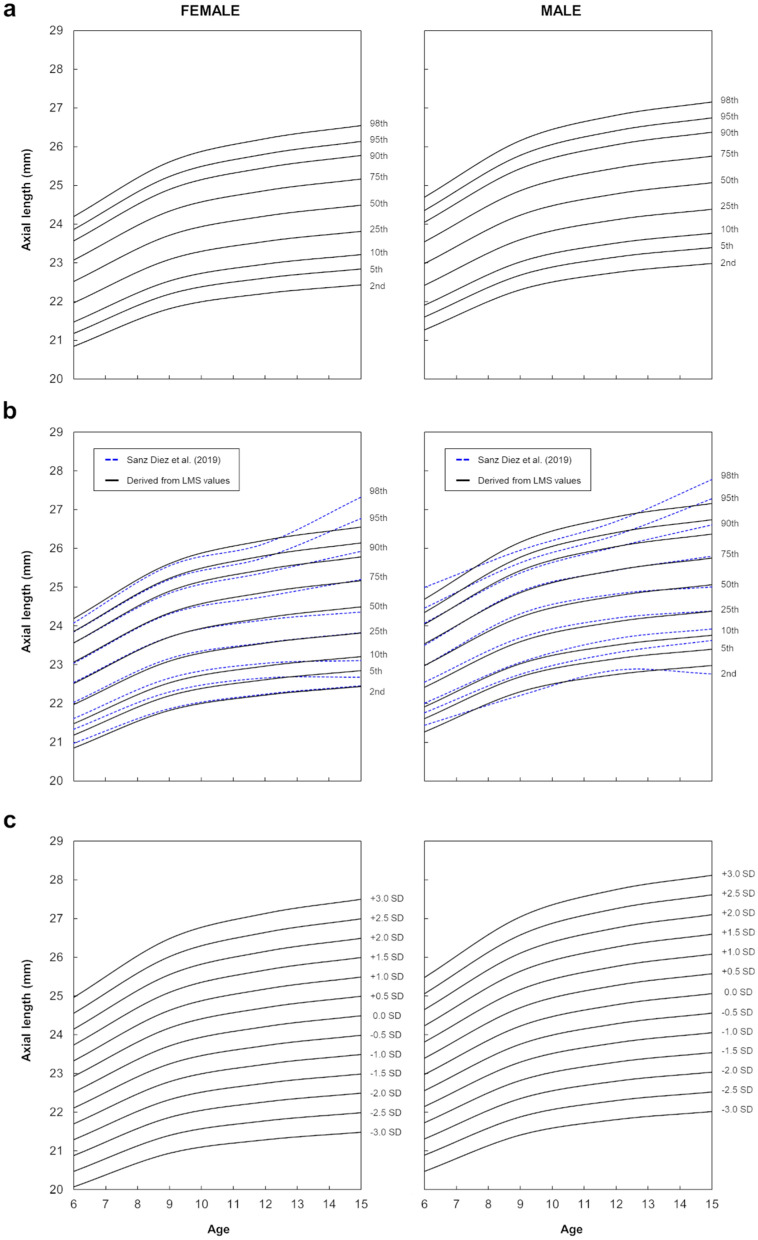
Growth curves of axial length derived from the LMS values. Female group (left) and male group (right). (**a**) Percentile curves of axial length derived from the LMS values. Nine percentile curves are displayed: 2nd, 5th, 10th, 25th, 50th, 75th, 90th, 95th and 98th. (**b**) Comparison of the axial length percentile growth curves as originally published by Sanz Diez et al.^10^ (blue dashed line) with those derived from the LMS values (black solid line). (**c**) Z-score growth curves of axial length derived from the LMS values. Z-scores range from − 3 to + 3 in 0.5 SD steps.

Figure 1a and Table 2 exhibit the new percentile growth curves for axial length generated using the LMS parameters. In both genders, all percentiles trended upward with age. The higher the percentile rank, the greater the increase in axial length. From 6 to 15 years, the 2nd percentile had a total axial elongation of 1.59 mm (females) and 1.71 mm (males), while the 98th increased by 2.35 mm (females) and 2.46 mm (males). Concretely, in the female group, the 10th percentile increased by 1.74 mm (21.47 mm at 6 years of age and 23.21 mm at 15 years of age), the 50th percentile increased by 1.97 (22.52 mm at 6 years of age and 24.49 mm at 15 years of age), and the 95th percentile increased by 2.28 mm (23.86 mm at 6 years of age and 26.14 mm at 15 years of age). In the male group, the 10th percentile increased by 1.85 mm (21.91 mm at 6 years of age and 23.76 mm at 15 years of age), the 50th increased by 2.09 (22.98 mm at 6 years of age and 25.07 mm at 15 years of age), and the 95th percentile increased by 2.39 mm (24.35 mm at 6 years of age and 26.74 mm at 15 years of age). Considering all percentile cut-off values at 6 and 15 years, total axial elongation was statistically significant in both females and males (two-sample t-test: p < 0.01, t-value =  − 3.05, 95% CI [− 3.34 to − 0.60], for females; and p < 0.01, t-value =  − 3.17, 95% CI [− 3.48 to − 0.69], for males).

Percentile axial elongation rates trended positively but unevenly with age. From 6 to 9 years of age, in females, the elongation rate between the 2nd and 98th percentiles ranged from 0.97 to 1.42 mm (4.67–5.85%), while in males ranged from 1.04 to 1.46 mm (4.87–5.93%). In this age range, those percentiles below and above the median showed a significant increase in axial length (two-sample t-test: p = 0.03, t-value =  − 2.89, 95% CI [− 1.92 to − 0.16], for females; and p = 0.02, t-value =  − 3.00, 95% CI [− 2.00 to − 0.20], for males). From 9 years onwards, the older the child, the lower the axial elongation rate. In the range 9–12 years, the elongation rate between the 2nd and 98th percentiles ranged from 0.40 to 0.60 mm (1.81–2.35%) in females. In males, ranged from 0.45 to 0.65 mm (2.00–2.50%). Compared to the 6–9-year range, this meant an average reduction in elongation rate of 61% in females and 58% in males at all percentiles. In the range 12–15 years, the elongation rate between the 2nd and 98th percentiles ranged from 0.22 to 0.34 mm (1.00–1.29%) in females, while ranged from 0.23 to 0.34 mm (1.02–1.28%) in males. Compared to the 6–9-year range this represented an average reduction in elongation rate of 79% in females and 78% in males at all percentiles. Specifically, between 12 and 15 years, no significant axial elongation was found in any of the generated percentiles (two-sample t-test: p = 0.69, t-value =  − 0.40, 95% CI [− 1.76 to 1.20], for females; and p = 0.69, t-value =  − 0.40, 95% CI [− 1.79 to 1.23], for males).

Supplementary Table 1 provides additional axial length percentiles (1st, 3rd, 97th and 99th) intended to facilitate comparison with other studies.

### Comparison with original growth curves (Sanz Diez et al.^10^)

Figure 1b shows the comparison between the originally published axial length growth curves^10^ and those derived from the LMS parameters. In females, between 6 and 15 years, the new percentile cut-off values ranged from 20.97 to 27.32 mm, while the originals ranged from 20.84 to 26.55 mm. The maximum discrepancies between the original percentiles and those newly generated appeared at the 95th and 98th percentiles at 15 years, where the differences were equal to 0.63 mm and 0.78 mm, respectively. Along the 50th and 90th percentiles, the highest differences between axial length curves were also found at 15 years of age, being 0.13 and 0.15 mm respectively. Along the 2nd, 5th and 10th percentiles, the maximum differences were on average 0.13 mm within the age range of 6 to 7 years. The minimum differences occurred along the 25th, 50th and 75th percentiles (mean difference: 0.05 ± 0.01 mm). In females, no significant differences between original and newly generated percentile curves were found (p > 0.77, t-value = [− 0.12, 0.29], df = 18; all superimposed percentile curves, two-sample t-test).

In males, between 6 and 15 years of age, the new percentile cut-off values ranged from 21.27 to 27.16 mm, while the originals ranged from 21.44 to 27.78 mm. The greatest discrepancies also appeared at 15 years at the 95th and 98th percentiles. Axial length differences were 0.54 mm for the 95th and 0.63 mm for the 98th percentile. Along the 2nd, 5th and 90th percentiles, the largest differences were also found at the age of 15 years (mean difference: 0.23 ± 0.01 mm). The smallest differences were also observed at the 25th, 50th and 75th percentiles (mean difference: 0.05 ± 0.03 mm). In males, no statistical differences were found between the original curves and those derived from the LMS parameters (p > 0.62, t-value = [− 0.02, 0.50], df = 18; all superimposed percentile curves, two-sample t-test).

### Z-scores

**Figure 2:**
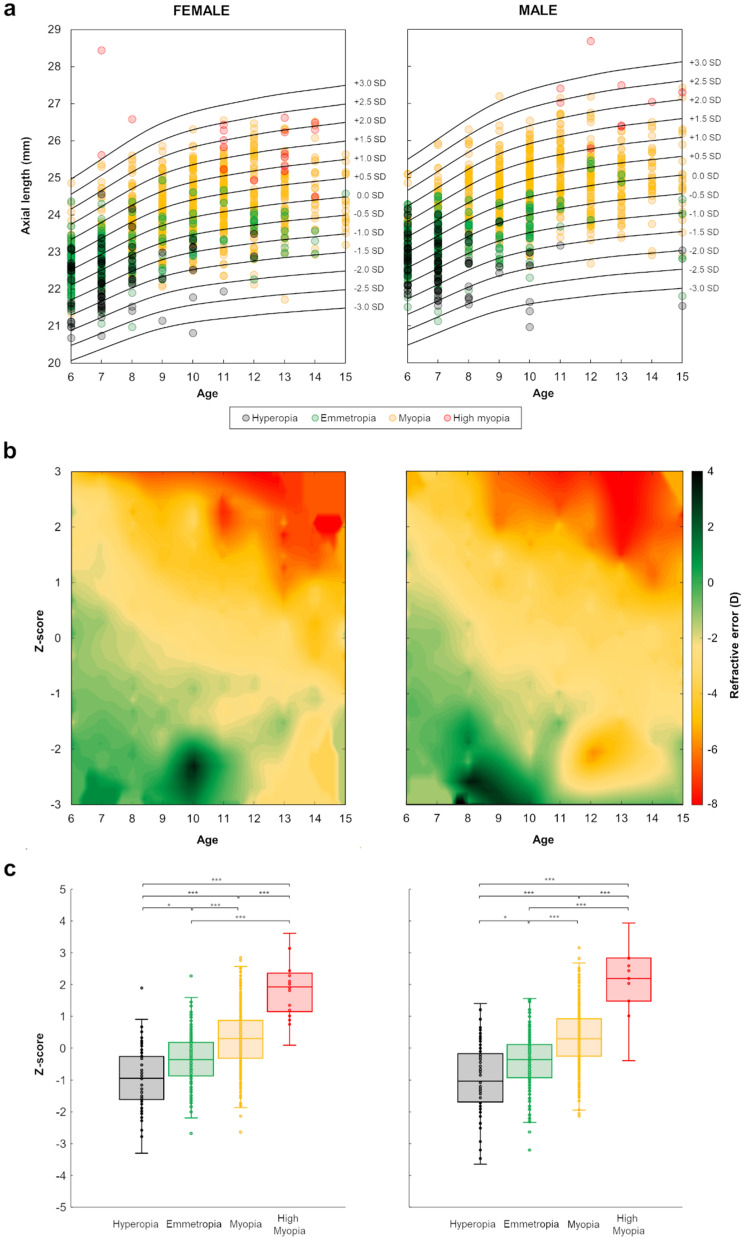
Z-scores based on axial length, age, and refraction. Female group (left) and male group (right). (**a**) Z-scores calculated from the second cross-sectional dataset and superimposed on the Z-score curves derived from the first cross-sectional dataset. (**b**) Refractive error distribution as a function of Z-scores and age. (**c**) Boxplots of Z-scores as a function of refractive condition. Statistical significance: *p < 0.05, **p < 0.01, ***p < 0.001.

Z-score curves are given for axial length based on age and gender (Fig. 1c, Table 3). From Eq. (2), the age-gender-specific LMS parameters provided in Tables 2 and 3 allow to calculate the Z-score corresponding to individual child’s axial length data. For example: a 7-year-old boy with an axial length of 24.51 mm would have a Z-score of 1.25. A 10-year-old girl with an axial length of 22.75 mm would have a Z-score of − 1.26. Accordingly, using age, gender, and axial length data from the second cross-sectional dataset, the corresponding Z-scores were calculated and graphically superimposed over the reference Z-scores curves for both gender groups (Fig. 2a). As depicted in Fig. 2a,b, there is a clear distribution of Z-scores according to the refractive status and age. From 6 to 15 years, in females, 82.35% of hyperopes and 64.71% of emmetropes were below the zero Z-score (0.0 SD or median) while 60% of myopes and 100% of high myopes were above it. Particularly, in females, the refractive values of those myopes located below (− 1.49 ± 0.88D) and above (− 2.49 ± 1.36D) the median differed statistically (p < 0.001, Mann–Whitney test). In males, 80.56% of hyperopes and 68.75% of emmetropes were below the 0.0 SD curve, whereas 64.14% of myopes and 90.91% of high myopes were above it. In males, refractive errors were also significantly different between those myopes below (− 1.60 ± 0.93D) and above (− 2.37 ± 1.29D) the 0.0 SD curve (p < 0.001, Mann–Whitney test).

Specifically, the most positive refractive values were in the range of 6 to 10 years of age and below 0.0 SD (Fig. 2b). In contrast, the most negative refractive values were mostly observed from 12 years and above + 1.0 SD curve (Fig. 2b). In both genders, the greater the Z-score rank, the higher the myopic refraction. In fact, Z-scores differed significantly based on refractive error (Fig. 2c). On average, Z-scores in females were − 0.94 ± 0.96 (hyperopes), − 0.34 ± 0.75 (emmetropes), 0.30 ± 0.90 (myopes) and 2.03 ± 1.36 (high myopes) (χ^2^ = 179.25, p < 0.001; Kruskal–Wallis test followed by Dunn–Sidak post-hoc test). In males, mean Z-scores were − 0.94 ± 1.06 (hyperopes), − 0.40 ± 0.81 (emmetropes), 0.33 ± 0.87 (myopes) and 2.25 ± 1.38 (high myopes) (χ^2^ = 181.45, p < 0.001; Kruskal–Wallis test followed by Dunn–Sidak post-hoc test).

### Z-scores as a predictor variable

To assess the predictive role of Z-scores on axial and refractive development, the longitudinal dataset (226 children) was employed. Age, gender, and axial length data at the first visit were used to determine the Z-scores. A linear regression analysis between Z-scores (first visit) and axial length values (third visit) revealed a significant direct relationship in females (F(1,85) = 143.77, r = 0.79, p < 0.001) and males (F(1,128) = 164.43, r = 0.75, p < 0.001). The linear regression analysis between Z-scores (first visit) and refractive errors at the third visit showed a significant negative correlation in females (F(1,85) = 56.53, r =  − 0.63, p < 0.001) and males (F(1,128) = 40.66, r =  − 0.49, p < 0.001). In contrast, the total change in axial length and refractive error (difference between first and third visits) were not associated with the Z-scores, in females (axial length: F(1,85) = 0.31, r =  − 0.06, p = 0.58 and refractive error: F(1,85) = 1.51, r =  − 0.13, p = 0.22) and males (axial length: F(1,128) = 0.66, r = 0.07, p = 0.42 and refractive error: F(1,128) = 0.01, r = 0.01, p = 0.95). The lower the Z-scores at the first visit, the lower the axial length and the more positive the refractive errors at the third visit.

A multinomial logistic regression was performed to predict the refractive condition at third visit (hyperopia, emmetropia, myopia or high myopia) from the first visit Z-scores. The final model significantly predicted the dependent variable better than the intercept-only model alone in females (χ^2^(3) = 18.47, p < 0.001) and males (χ^2^(3) = 14.42, p = 0.002). Particularly, Z-scores were able to significantly discriminate high myopia from the other three types of refractive errors developed at the third visit (χ^2^(3) = 26.96, p < 0.001). For each one unit increase in Z-score, the log-odds of an individual falling into the hyperopia, emmetropia or myopia categories (relative to the high myopia category) is predicted to decrease by 3.12, 1.94 and 1.22 units, respectively. Moreover, as reported in the odds ratio (OR) column, with increasing Z-score values, the odds of falling into the hyperopia, emmetropia and myopia categories as changing by a factor of 0.044, 0.144 and 0.297 (Table 4). In both genders, those individuals classified as high myopes at the third visit were more likely to have higher Z-scores at the first visit (female group: β = 4.02, Std. error = 2.03, χ^2^(1) = 3.92, p < 0.05; male group: β = 1.46, Std. error = 0.65, χ^2^(1) = 5.07, p = 0.02).

**Table 4:** Multinomial logistic regression model used to predict high myopia at third visit given Z-score from first visit.

		β	Standard error	Chi-square(df = 1)	OR (95% CI)	p-value
Hyperopia	Intercept	− 1.30	1.02	1.63	–	0.20
	Z-scores	− 3.12	0.77	16.46	0.044 (0.01–0.20)	< 0.001
Emmetropia	Intercept	0.50	0.65	0.59	–	0.44
	Z-scores	− 1.94	0.63	9.52	0.144 (0.04–0.49)	0.002
Myopia	Intercept	3.73	0.53	50.10	–	< 0.001
	Z-scores	− 1.22	0.47	6.70	0.297 (0.12–0.74)	0.01

The reference category is high myopia.

The ROC analysis revealed Z-scores as a good predictive factor for high myopia, with an area under the curve of 0.84 for females (standard error: 0.04; 95% CI 0.76–0.92, p < 0.001) and 0.76 for males (standard error: 0.12; 95% CI 0.52–0.99, p = 0.03). As mentioned earlier for both genders, high myopia developed in most of those children whose Z-score (calculated from axial length) at the first visit was near to or above + 1.0 SD. In females, the Z-score cut-off value of + 0.88 SD showed sensitivity and specificity of 100.00% and 83.70%, respectively. In males, the Z-score cut-off value of + 0.91 SD exhibited sensitivity of 71.40% and specificity of 89.40%.

## Discussion

In this paper we have illustrated the use of the LMS method for the development of axial length growth curves in a Chinese school-age population. The LMS method has been used to calculate the LMS parameters, from which the percentile and Z-score growth curves for axial length have been generated. Additionally, a comparison was made between the growth curves derived from the LMS parameters and the original curves calculated from the same study population^10^.

Interestingly, we have observed a close agreement between the percentiles derived from the LMS parameters and those originally developed^10^. Both methodologies showed comparable percentile trends with larger discrepancies at ages 14 and 15. Percentile curves have exhibited an increase in axial length with age. Particularly, the increase in axial length was percentile-dependent and inconsistent across different ages, with the youngest individuals increasing the fastest. These findings agree with the trends reported by other percentile growth studies. However, the rate of axial elongation differs considerably from those reported in European population studies^11,13,15^. Interestingly, the largest discrepancies between cohorts occur in the 6 to 9-year age range. In fact, this is consistent with the annual changes observed within this range, being on average 0.19 mm/year in the European cohort, while 0.41 mm/year in the Asian. Similar axial elongation rates have been reported by other authors, who have showed variances based on the refractive condition^8,9^. This emphasizes the importance of a close monitoring of the ocular components during the school years to carefully supervise possible structural and refractive changes^26,27^.

Given the LMS parameters calculated from the reference population, Z-score of any child can be calculated from their age and axial length. Z-scores showed a defined distribution pattern according to individuals’ refractive condition and age. Considering the median as a reference, all hyperopic and emmetropic subjects were below it. In contrast, myopic and high myopic subjects were above it. Z-scores were found to be good predictors for high myopia. Multinomial analysis revealed Z-scores to be able to discriminate high myopia from the other refractive conditions. Moreover, ROC analysis revealed good performance values. These findings suggest the feasibility of Z-scores in the study of ocular component growth patterns, since are based on a reference population and can be studied as a continuous variable^16^.

Despite the reported findings, the main limitation of the study is the failure to consider myopia risk factors in the generation of the percentile and Z-score growth curves. Applying the effect of risk factors could result in improved accuracy. Another limitation is the extrapolation of results to other populations. The development and application of axial length growth curves should consider the geographic area and ethnicity of the study population. Axial length percentile growth curves for different geographic regions are needed to define global strategies for myopia prevention.

In summary, we provide a demonstration of the LMS method on ocular axial length data. While the LMS method has been widely applied in the anthropometric assessment for children and adolescents, its implementation has not been extended to the analysis of ocular axial length growth patterns. In this research we have established for the first time the LMS parameters for axial length, from which we have generated axial length growth curves comparable to those originally published. Additionally, we have observed the practicality and helpfulness of the LMS methodology for the calculation of axial length Z-score curves, which enable a more precise assessment of eye growth during childhood.

We believe, the comprehensive study of ocular growth patterns in school children necessitates the use of valid methodologies to generate percentile growth curves and Z-scores that can shed light on the field of vision science. These findings may assist organizations and governments in evaluating and designing appropriate myopia prevention programs for children and adolescents.

## Methods

### Study population and data collection

The current study is based on a secondary analysis of the population from a previously published prospective cross-sectional study of school-aged children from Wuhan, China^10^. Eye data was collected by the Wuhan Center for Adolescent Poor Vision Prevention and Control. A total of 14,760 individuals (7133 girls and 7627 boys) were included in the study. Three datasets were used: two cross-sectional datasets and one longitudinal dataset. The first cross-sectional dataset was composed of 6054 girls (9.99 ± 2.47 years) and 6500 boys (9.90 ± 2.48 years) and was used to generate the growth curves for axial length, which served as a reference. The second cross-sectional set of data included 987 girls (9.36 ± 2.39 years) and 993 boys (9.32 ± 2.45 years) and assisted in showing the methodology implementation and in observing the distribution of generated growth curves as a function of refractive errors and axial length data. The longitudinal dataset consisted of 226 children (92 girls and 134 boys) with a total of three appointments over time and a mean time difference between the first and the third appointment of 2.67 ± 2.96 years for girls and 2.57 ± 2.77 years for boys. The longitudinal dataset was used to explore the efficiency of the newly calculated curves in predicting high myopic values.

Cycloplegic spherical refractive error data was obtained using the Topcon CV-3000 autorefractometer (Topcon, Tokyo, Japan). Axial length data was measured using a non-contact optical biometer, Lenstar LS900 (Haag-Streit AG, Koeniz, Switzerland). For more information on the study population and data acquisition, see Sanz Diez et al.^10^.

The study and data acquisition were approved by the Ethical Committee of the Wuhan Center for Adolescent Poor Vision Prevention and Control. Written and oral information was given, after which written informed consent was obtained from all participants or legal representatives. The study was conducted in accordance with the Declaration of Helsinki.

### Percentile curves based on LMS method

Considering the purposes of the current study and the percentile range chosen in groundbreaking studies on axial length percentiles^10,11^, nine percentile curves (2nd, 5th, 10th, 25th, 50th, 75th, 90th, 95th and 98th) were generated. Here, percentiles were constructed through the LMS method, described by Cole^18–20^. The LMS method is a mathematical model that allows to fit longitudinal and cross-sectional anthropometric data, ocular axial length here, to obtain normalized percentile curves^20,21^. LMS method summarizes the distribution of the variable of interest according to age, based on three parameters or curves: L (λ), M (µ), and S (σ). These three parameters indicate the power in the Box-Cox transformation for the skewness adjustment (L), the median (M), and the generalized coefficient of variation (S) for each annual measurement. Assuming the residuals follow a normal distribution and by using the three estimated parameters (L(x), M(x) and S(x)) for each age (x), axial length values can be converted into percentiles and Z-scores. Percentiles at age (x) can be calculated from Eq. (1), where C_100α_(x) is the 100_α_-th percentile rank and Z_α_ is the desired percentile in standard deviation units.

1 $${C}_{100\alpha }\left(x\right)=\left\{\genfrac{}{}{0pt}{}{M\left(x\right){\left[1+L\left(x\right)S\left(x\right){Z}_{\alpha }\right]}^{\frac{1}{L\left(x\right)}} ;\quad L(x) \ne 0}{\begin{array}{l} \\ \\ M\left(x\right)\mathrm{exp}\left[S\left(x\right){Z}_{\alpha }\right] ;\quad L\left(x\right) = 0\end{array}}\right..$$

The corresponding Z-scores at age (x) can be obtained from Eq. (2), where y indicates the individual axial length measurement.

2 $$Z=\left\{\genfrac{}{}{0pt}{}{\frac{{[\frac{y}{M\left(x\right)}]}^{L\left(x\right)}-1}{S\left(x\right)L\left(x\right)} ;\quad L(x) \ne 0}{\begin{array}{l} \\ \\ \frac{\mathrm{ln}[\frac{y}{M\left(x\right)}]}{S\left(x\right)} ;\quad L(x) = 0\end{array}}\right..$$

Based on the maximum penalized likelihood methodology, LMS curves can be fitted as cubic splines by non-linear regression^18^. The degree of smoothness is defined by three smoothing parameters or equivalent degrees of freedom (edf). Each edf indicates the complexity of each L, M and S curve. A more detailed description of the LMS method can be found elsewhere^18–20^.

### Curve modelling

LMSchartmaker Pro software (version 2.54, Medical Research Council, UK) was used to calculate the LMS parameters and subsequently construct the axial length growth curves based on the LMS method. LMSchartmaker is a software developed by Pan and Cole to fit smooth percentile curves to reference data using the LMS method^28^.

Under the software protocol, curve modelling involves selecting the appropriate age scale and choosing the proper edf values to optimize the L, M and S curves. Deviance measure is the benchmark for model fitting and curves optimization: the smaller the deviance measure, the better the optimization of the L, M and S curves. As a deviance measure, this study followed the authors’ recommendations, and therefore Schwarz Bayesian Criterion (SBC) was used as a deviance measure for model fitting^28^. A more complete information of the software modelling statistics can be seen elsewhere^28^. Following Sanz Diez et al., axial length growth curves were computed considering both gender and age^10^.

### Statistical analysis

Statistical analyses were performed using the MATLAB R2020a statistical toolbox (MathWorks, USA) and SPSS statistical software package, v-27.0 (SPSS, Chicago, Illinois, USA). Data distribution was inspected visually (frequency distribution and quantile–quantile plot) and statistically (Kolmogorov–Smirnov test). Statistical analyses were done using the appropriate tests depending on the data distribution. Differences were considered statistically significant when p < 0.05. Results are provided as mean ± standard deviations. Multinomial logistic regression was performed, with refractive state condition as dependent outcome variable. Cox and Snell’s, Nagelkerke’s and McFadden’s goodness-of-fit tests were used to evaluate the fit of the model. Likelihood ratio tests were used to assess the contribution of the independent variable to the model. Receiver operating characteristic (ROC) analysis was conducted to assess the diagnostic performance of Z-scores as a prediction model. ROC area of 1.0 indicates an ideal test, while an area of 0.5 describes an inaccurate test.

Comparisons of percentile curves of axial length data between original growth curves^10^ and growth curves derived from the LMS method were performed in two ways. First, a comparison of the original and the newly calculated percentiles was prepared by means of a graphical superimposition of each of them. Second, differences between the percentile cut-off values of both growth curves were assessed by the Student's t-test at all ages.

To cluster the results according to the spherical refractive error (SR), four conditions were used: hyperopia (SR >  + 0.50D), emmetropia (− 0.50 < SR ≤  + 0.50D), myopia (SR ≤  − 0.50D), and high myopia (SR ≤  − 6.00D).

## Supplementary Information

Supplementary Table 1.

## Data Availability

The datasets generated during the current study are available from the corresponding author on reasonable request.
