# Supplementary material for: LMS parameters, percentile, and Z-score growth curves for axial length in Chinese schoolchildren in Wuhan
Source: Sci Rep. 2022 Mar 22;12:4850. doi: 10.1038/s41598-022-08907-5 (PMC8941183; doi:10.1038/s41598-022-08907-5)
Supplement: Supplementary file 1 — Supplementary Table 1. [file 41598_2022_8907_MOESM1_ESM.docx]

**Supplementary Table 1.** LMS parameters and axial length percentiles in millimeters, as a function of age and gender. Percentile’s 1^st^, 3^rd^, 97^th^ and 99^th^ have been added in this supplementary table to facilitate the use and comparison of the reported data with other similar studies.

| **Female** | | | | | | | | | | | | | | | | |
| --- | --- | --- | --- | --- | --- | --- | --- | --- | --- | --- | --- | --- | --- | --- | --- | --- |
|  | | | | **Percentiles (Axial length in mm)** | | | | | | | | | | | | |
| **Age** | **L** | **M** | **S** | **1st** | **2nd** | **3rd** | **5th** | **10th** | **25th** | **50th** | **75th** | **90th** | **95th** | **97th** | **98th** | **99th** |
| 6 | 1.0 | 22.52 | 0.0362 | 20.62 | 20.84 | 20.98 | 21.18 | 21.47 | 21.97 | 22.52 | 23.07 | 23.56 | 23.86 | 24.05 | 24.19 | 24.42 |
| 7 | 1.0 | 22.94 | 0.0372 | 20.96 | 21.19 | 21.34 | 21.54 | 21.85 | 22.37 | 22.94 | 23.52 | 24.04 | 24.35 | 24.55 | 24.70 | 24.93 |
| 8 | 1.0 | 23.37 | 0.0381 | 21.29 | 21.54 | 21.69 | 21.90 | 22.22 | 22.77 | 23.37 | 23.97 | 24.51 | 24.83 | 25.04 | 25.20 | 25.44 |
| 9 | 1.0 | 23.71 | 0.0389 | 21.57 | 21.82 | 21.98 | 22.19 | 22.53 | 23.09 | 23.71 | 24.34 | 24.90 | 25.23 | 25.45 | 25.61 | 25.86 |
| 10 | 1.0 | 23.94 | 0.0395 | 21.74 | 22.00 | 22.16 | 22.38 | 22.73 | 23.30 | 23.94 | 24.58 | 25.15 | 25.49 | 25.72 | 25.88 | 26.14 |
| 11 | 1.0 | 24.09 | 0.0399 | 21.85 | 22.12 | 22.28 | 22.51 | 22.86 | 23.44 | 24.09 | 24.73 | 25.32 | 25.67 | 25.89 | 26.06 | 26.32 |
| 12 | 1.0 | 24.21 | 0.0402 | 21.95 | 22.21 | 22.38 | 22.61 | 22.96 | 23.55 | 24.21 | 24.87 | 25.46 | 25.81 | 26.04 | 26.21 | 26.48 |
| 13 | 1.0 | 24.32 | 0.0405 | 22.03 | 22.30 | 22.47 | 22.70 | 23.06 | 23.66 | 24.32 | 24.98 | 25.58 | 25.94 | 26.17 | 26.34 | 26.61 |
| 14 | 1.0 | 24.41 | 0.0407 | 22.10 | 22.37 | 22.54 | 22.77 | 23.13 | 23.74 | 24.41 | 25.08 | 25.68 | 26.04 | 26.27 | 26.45 | 26.72 |
| 15 | 1.0 | 24.49 | 0.0409 | 22.16 | 22.43 | 22.61 | 22.84 | 23.21 | 23.82 | 24.49 | 25.17 | 25.78 | 26.14 | 26.38 | 26.55 | 26.82 |
| **Male** | | | | | | | | | | | | | | | | |
|  | | | | **Percentiles (Axial length in mm)** | | | | | | | | | | | | |
| **Age** | **L** | **M** | **S** | **1st** | **2nd** | **3rd** | **5th** | **10th** | **25th** | **50th** | **75th** | **90th** | **95th** | **97th** | **98th** | **99th** |
| 6 | 1.0 | 22.98 | 0.0363 | 21.04 | 21.27 | 21.41 | 21.61 | 21.91 | 22.42 | 22.98 | 23.54 | 24.05 | 24.35 | 24.55 | 24.70 | 24.92 |
| 7 | 1.0 | 23.42 | 0.0372 | 21.40 | 21.63 | 21.78 | 21.99 | 22.31 | 22.84 | 23.42 | 24.01 | 24.54 | 24.86 | 25.06 | 25.21 | 25.45 |
| 8 | 1.0 | 23.87 | 0.0381 | 21.75 | 22.00 | 22.16 | 22.37 | 22.70 | 23.25 | 23.87 | 24.48 | 25.03 | 25.36 | 25.57 | 25.73 | 25.98 |
| 9 | 1.0 | 24.23 | 0.0388 | 22.05 | 22.30 | 22.46 | 22.69 | 23.03 | 23.60 | 24.23 | 24.86 | 25.43 | 25.78 | 26.00 | 26.16 | 26.42 |
| 10 | 1.0 | 24.48 | 0.0393 | 22.24 | 22.50 | 22.67 | 22.89 | 23.24 | 23.83 | 24.48 | 25.12 | 25.71 | 26.06 | 26.28 | 26.45 | 26.71 |
| 11 | 1.0 | 24.64 | 0.0397 | 22.37 | 22.64 | 22.81 | 23.04 | 23.39 | 23.98 | 24.64 | 25.30 | 25.89 | 26.25 | 26.48 | 26.65 | 26.92 |
| 12 | 1.0 | 24.78 | 0.0400 | 22.48 | 22.75 | 22.92 | 23.15 | 23.51 | 24.11 | 24.78 | 25.45 | 26.05 | 26.41 | 26.64 | 26.81 | 27.08 |
| 13 | 1.0 | 24.90 | 0.0402 | 22.57 | 22.84 | 23.01 | 23.25 | 23.61 | 24.22 | 24.90 | 25.57 | 26.18 | 26.54 | 26.78 | 26.95 | 27.22 |
| 14 | 1.0 | 24.98 | 0.0404 | 22.63 | 22.91 | 23.08 | 23.32 | 23.69 | 24.30 | 24.98 | 25.66 | 26.28 | 26.64 | 26.88 | 27.05 | 27.33 |
| 15 | 1.0 | 25.07 | 0.0406 | 22.70 | 22.98 | 23.15 | 23.39 | 23.76 | 24.38 | 25.07 | 25.75 | 26.37 | 26.74 | 26.98 | 27.16 | 27.43 |
